# 2-{(*E*)-[(2-Hy­droxy-1-phenyl­eth­yl)imino]­meth­yl}-4-[(*E*)-(4-methyl­phen­yl)diazen­yl]phenol

**DOI:** 10.1107/S2414314625005991

**Published:** 2025-07-08

**Authors:** Mohammad H. Ati, Basil A. Saleh, Adnan J. M. Al-Fartosy, Gamal A. El-Hiti, Benson M. Kariuki

**Affiliations:** a Department of Chemistry, College of Science, University of Basrah, Basrah 61004, Iraq; b Department of Optometry, College of Applied Medical Sciences, King Saud, University, Riyadh 11433, Saudi Arabia; c School of Chemistry, Cardiff University, Main Building, Park Place, Cardiff CF10, 3AT, United Kingdom; Katholieke Universiteit Leuven, Belgium

**Keywords:** crystal structure, Schiff base

## Abstract

In the title compound, C_22_H_21_N_3_O_2_, an intra­molecular O—H⋯N hydrogen bond is observed between the phenol and methanimine groups of the mol­ecule. The 2-phenyl­ethan-1-ol part is disordered over two orientations [occupancies 0.566 (17) and 0.434 (17)]. In the crystal, disordered inter­molecular O—H⋯O hydrogen bonds between adjacent mol­ecules form chains parallel to the *b* axis.

## Abstract 2

Intra­molecular O1—H1⋯N3 hydrogen bonding occurs in the title mol­ecule and inter­molecular O—H⋯O hydrogen bonds form chains parallel to the *b* axis.

## Graphical abstract

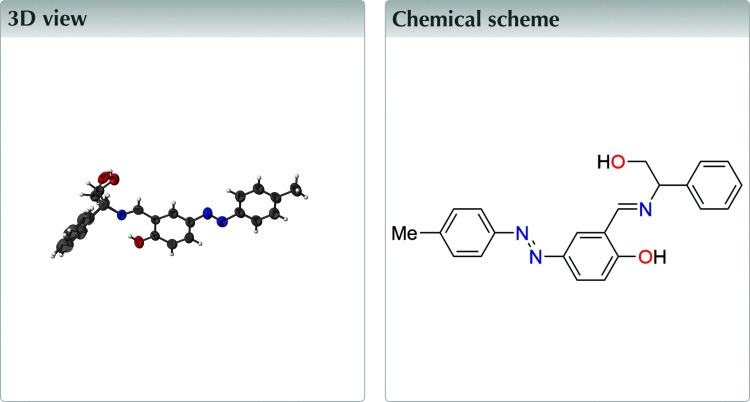

## Structure description

Primary amines react with azo compounds, along with aldehydes or ketones, to yield azo-Schiff bases (Su *et al.*, 2015 ▸). Azo-Schiff bases serve as chelating ligands in coordination chemistry, where they form complexes with different metal ions that can be applied in catalysis and materials science (Kargar *et al.*, 2022 ▸). Studies in the pharmaceutical sector have shown that azo-Schiff bases possess significant biological activities, including anti­microbial (da Silva *et al.*, 2011 ▸), anti­oxidant (Hameed *et al.*, 2017 ▸), and anti­cancer (El-Sonbati *et al.*, 2015 ▸) effects. Their potential for inter­action with biological targets like enzymes and DNA has paved the way for new drug development approaches (Kaswan, 2023 ▸). Moreover, the ability of the azo group to function as a free radical scavenger boosts its potential in addressing oxidative stress-related disorders (Su *et al.*, 2015 ▸). This work details the synthesis and crystal structure of an azo-Schiff base.

**Figure 1:**
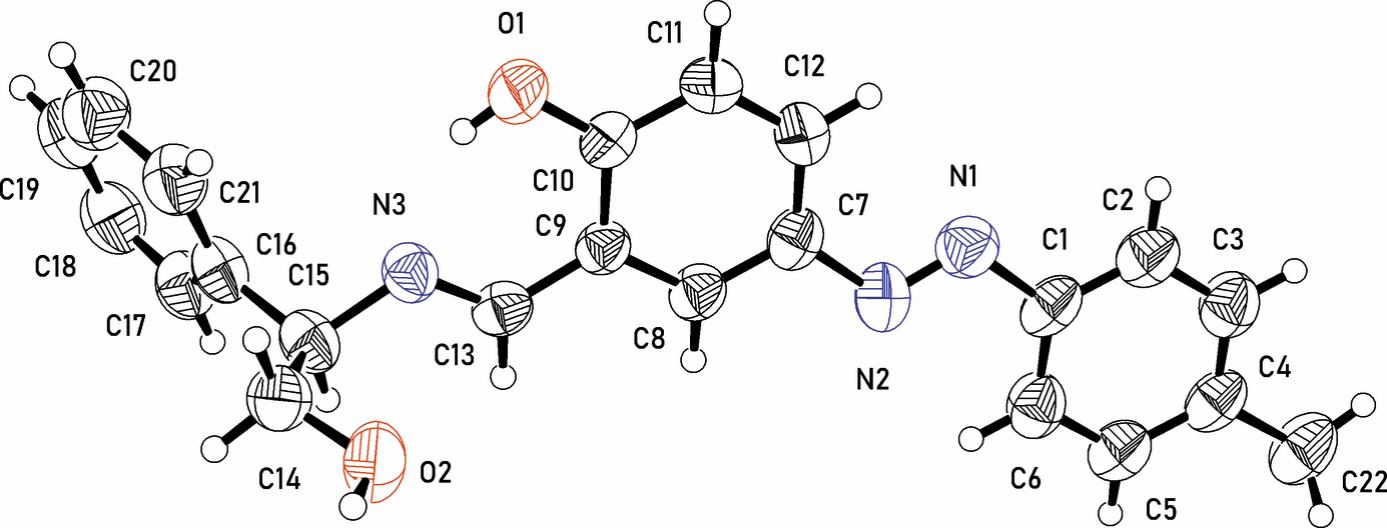
An *ORTEP* representation of the title compound showing 50% probability ellipsoids. Only the major component of the disordered 2-phenyl­ethan-1-ol segment is shown.

The asymmetric unit of the crystal structure comprises one mol­ecule of the title compound (Fig. 1 ▸). The mol­ecule consists of a (tolyl­diazen­yl)phenol segment (C1-C12/C22/N1/N2/O1) linked to a 2-phenyl­ethan-1-ol segment (C14–C21/O2) by a methanimine group (C13, N3). The (tolyl­diazen­yl)phenol and methanimine segments are almost co-planar [torsion angle C8—C9—C13—N3 is 178.9 (3)°]. The 2-phenyl­ethan-1-ol part is disordered over two orientations [occupancies 0.566 (17) for component containing O2, and 0.434 (17) for component containing O2*A*]. The inter­planar angle between the rings C1–C6 and C7–C12 is 1.39 (18)°, between rings C1–C6 and C16–C21 62.0 (5)°, and between rings C1–C6 and C16*A*–C21*A* 62.4 (5)°.

An intra­molecular O1—H1⋯N3 hydrogen bond occurs between the phenol and methanimine groups (Table 1 ▸). The two positions of the 2-phenyl­ethan-1-ol moiety allow two alternative O—H⋯O hydrogen bonds to be formed with adjacent mol­ecules in the crystal packing (Table 1 ▸), one of which forms chains of mol­ecules propagating parallel to the *b* axis. The other hydrogen bond links pairs of molecules within the chain perpendicular to the direction of chain propagation.

## Synthesis and crystallization

A solution of 4-toluidine (0.214 g, 2 mmol) in concentrated HCl (2 ml) and H_2_O (5 ml) was cooled to 273–278 K. Sodium nitrite (0.14 g, 2 mmol) in H_2_O (0.5 ml) was added dropwise over 10 minutes. The mixture was stirred for 30 minutes at 273–278 K, followed by the addition of salicyl­aldehyde (0.244 g, 2 mmol), H_2_O (4 ml), NaOH (0.08 g, 2.0 mmol), and Na_2_CO_3_ (0.74 g, 7.0 mmol). The mixture was then stirred for 1 h at 273–278 K. The crude azo product was filtered, washed with H_2_O, and dried at 298 K under vacuum. A solution of d-phenyl­glycinol (0.137 g, 1.0 mmol) in MeOH (20 ml) was added to a solution of the azo product (0.240 g, 1.0 mmol) in MeOH (20 ml). The mixture was refluxed for 3 h, and the solid obtained was removed by filtration, dried, and crystallized from ethanol solution to give yellow needles of the title compound in 54.1% yield, m.p. 463–465 K. (KBr) ν (cm^−1^): 3178, 2979, 1615, 1495, 1372. ^1^H NMR (400 MHz, DMSO; δ p.p.m.): 14.44 (*s*, 1H), 8.80 (*s*, 1H), 8.11 (*d*, *J* = 2.6 Hz, 1H), 7.92 (*dd*, *J* = 8.9, 2.6 Hz, 1H), 7.74 (*d*, *J* = 8.1 Hz, 2H), 7.45–7.32 (*m*, 7H), 7.01 (*d*, *J* = 8.9 Hz, 1H), 5.22 (*s*, 1H), 4.57 (*dd*, *J* = 8.3, 4.4 Hz, 1H), 3.73 (*m*, 2H), 2.39 (*s*, 3H). ^13^C NMR (100 MHz, DMSO; δ p.p.m.): 166.6, 165.9, 150.5, 144.2, 141.2, 140.2, 130.4, 129.7, 129.1, 128.1, 127.5, 126.5, 121.7, 119.2, 118.5, 74.0, 66.3, 21.5. The absolute configuration of C15 is *R*.

## Refinement

Crystal data, data collection and structure refinement details are summarized in Table 2 ▸. The 2-phenyl­ethan-1-ol part of the mol­ecule is disordered and was modeled as two components with occupancies refining to 0.566 (17) and 0.434 (17).

## Supplementary Material

Crystal structure: contains datablock(s) I. DOI: 10.1107/S2414314625005991/vm4068sup1.cif

Structure factors: contains datablock(s) I. DOI: 10.1107/S2414314625005991/vm4068Isup2.hkl

Supporting information file. DOI: 10.1107/S2414314625005991/vm4068Isup3.cml

CCDC reference: 2469543

Additional supporting information:  crystallographic information; 3D view; checkCIF report

## Figures and Tables

**Table 1:** Hydrogen-bond geometry (Å, °)

*D*—H⋯*A*	*D*—H	H⋯*A*	*D*⋯*A*	*D*—H⋯*A*
O2—H2*A*⋯O1^i^	0.82	2.00	2.745 (10)	151
O1—H1⋯N3	0.82	1.88	2.605 (4)	147
O2*A*—H2*B*⋯O2^ii^	0.82	2.14	2.88 (2)	149

Symmetry codes: (i) 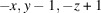; (ii) 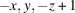.

**Table 2:** Experimental details

Crystal data	
Chemical formula	C_22_H_21_N_3_O_2_
*M*_r_	359.42
Crystal system, space group	Monoclinic, *I*2
Temperature (K)	293
*a*, *b*, *c* (Å)	14.7749 (8), 6.0051 (3), 22.3625 (12)
β (°)	108.080 (6)
*V* (Å^3^)	1886.14 (18)
*Z*	4
Radiation type	Mo *K*α
μ (mm^−1^)	0.08
Crystal size (mm)	0.28 × 0.25 × 0.20

Data collection	
Diffractometer	SuperNova, Dual, Cu at home/near, Atlas
Absorption correction	Gaussian (*CrysAlis PRO*; Rigaku OD, 2024 ▸)
*T*_min_, *T*_max_	0.514, 1.000
No. of measured, independent and observed [*I* > 2σ(*I*)] reflections	15526, 4696, 3546
*R*_int_	0.024
(sin θ/λ)_max_ (Å^−1^)	0.698

Refinement	
*R*[*F*^2^ > 2σ(*F*^2^)], *wR*(*F*^2^), *S*	0.054, 0.148, 1.07
No. of reflections	4696
No. of parameters	318
No. of restraints	450
H-atom treatment	H-atom parameters constrained
Δρ_max_, Δρ_min_ (e Å^−3^)	0.28, −0.17
Absolute structure	Flack *x* determined using 1258 quotients [(*I*^+^)−(*I*^−^)]/[(*I*^+^)+(*I*^−^)] (Parsons et al., 2013 ▸)
Absolute structure parameter	0.1 (5)

Computer programs: *CrysAlis PRO CCD* (Rigaku OD,2024 ▸)), *CrysAlis PRO* (Rigaku OD, 2023 ▸), *SHELXT* (Sheldrick, 2015*a* ▸), *SHELXL2019/2* (Sheldrick, 2015*b* ▸), *Mercury* (Macrae *et al.*, 2020 ▸) and *publCIF* (Westrip, 2010 ▸).

## full crystallographic data

### 2-{(E)-[(2-Hydroxy-1-phenylethyl)imino]methyl}-4-[(E)-(4-methylphenyl)diazenyl]phenol Crystal data

**Table:**

C_22_H_21_N_3_O_2_	*F*(000) = 760
*M**_r_* = 359.42	*D*_x_ = 1.266 Mg m^−^^3^
Monoclinic, *I*2	Mo *K*α radiation, λ = 0.71073 Å
*a* = 14.7749 (8) Å	Cell parameters from 6820 reflections
*b* = 6.0051 (3) Å	θ = 2.9–27.5°
*c* = 22.3625 (12) Å	µ = 0.08 mm^−^^1^
β = 108.080 (6)°	*T* = 293 K
*V* = 1886.14 (18) Å^3^	Block, orange
*Z* = 4	0.28 × 0.25 × 0.20 mm

### 2-{(E)-[(2-Hydroxy-1-phenylethyl)imino]methyl}-4-[(E)-(4-methylphenyl)diazenyl]phenol Data collection

**Table:**

SuperNova, Dual, Cu at home/near, Atlas diffractometer	3546 reflections with *I* > 2σ(*I*)
Detector resolution: 10.5082 pixels mm^-1^	*R*_int_ = 0.024
ω scans	θ_max_ = 29.7°, θ_min_ = 2.8°
Absorption correction: gaussian (CrysAlisPro; Rigaku OD, 2024)	*h* = −20→20
*T*_min_ = 0.514, *T*_max_ = 1.000	*k* = −8→7
15526 measured reflections	*l* = −30→30
4696 independent reflections	

### 2-{(E)-[(2-Hydroxy-1-phenylethyl)imino]methyl}-4-[(E)-(4-methylphenyl)diazenyl]phenol Refinement

**Table:**

Refinement on *F*^2^	Hydrogen site location: inferred from neighbouring sites
Least-squares matrix: full	H-atom parameters constrained
*R*[*F*^2^ > 2σ(*F*^2^)] = 0.054	*w* = 1/[σ^2^(*F*_o_^2^) + (0.0466*P*)^2^ + 1.3125*P*] where *P* = (*F*_o_^2^ + 2*F*_c_^2^)/3
*wR*(*F*^2^) = 0.148	(Δ/σ)_max_ < 0.001
*S* = 1.07	Δρ_max_ = 0.28 e Å^−^^3^
4696 reflections	Δρ_min_ = −0.17 e Å^−^^3^
318 parameters	Absolute structure: Flack *x* determined using 1258 quotients [(*I*^+^)-(*I*^-^)]/[(*I*^+^)+(*I*^-^)] (Parsons et al., 2013)
450 restraints	Absolute structure parameter: 0.1 (5)

### 2-{(E)-[(2-Hydroxy-1-phenylethyl)imino]methyl}-4-[(E)-(4-methylphenyl)diazenyl]phenol Special details

**Table:**

Geometry. All esds (except the esd in the dihedral angle between two l.s. planes) are estimated using the full covariance matrix. The cell esds are taken into account individually in the estimation of esds in distances, angles and torsion angles; correlations between esds in cell parameters are only used when they are defined by crystal symmetry. An approximate (isotropic) treatment of cell esds is used for estimating esds involving l.s. planes.
Refinement. Non-hydrogen atoms were refined with anisotropic displacement parameters. In the final cycles of refinement, hydrogen atom geometry was idealized, and a riding model was used with *U*_iso_ set at 1.2 or 1.5 times the value of *U*_eq_ for the atom to which the hydrogen atoms are bonded.

### 2-{(E)-[(2-Hydroxy-1-phenylethyl)imino]methyl}-4-[(E)-(4-methylphenyl)diazenyl]phenol Fractional atomic coordinates and isotropic or equivalent isotropic displacement parameters (Å^2^)

**Table:**

	*x*	*y*	*z*	*U*_iso_*/*U*_eq_	Occ. (<1)
C1	0.6053 (2)	0.3897 (6)	0.62137 (17)	0.0589 (9)	
C2	0.6836 (3)	0.4712 (7)	0.66603 (18)	0.0658 (10)	
H2	0.681939	0.612038	0.682988	0.079*	
C3	0.7648 (3)	0.3448 (7)	0.68589 (19)	0.0678 (10)	
H3	0.817801	0.401195	0.716574	0.081*	
C4	0.7697 (2)	0.1358 (7)	0.66138 (17)	0.0613 (9)	
C5	0.6900 (3)	0.0521 (7)	0.61597 (18)	0.0647 (10)	
H5	0.692188	−0.088654	0.599117	0.078*	
C6	0.6068 (3)	0.1773 (7)	0.59551 (18)	0.0654 (10)	
H6	0.553136	0.121343	0.565295	0.078*	
C7	0.3730 (2)	0.5987 (6)	0.54591 (16)	0.0541 (8)	
C8	0.2943 (2)	0.5117 (6)	0.50225 (15)	0.0516 (7)	
H8	0.298040	0.370315	0.486231	0.062*	
C9	0.2087 (2)	0.6284 (5)	0.48102 (14)	0.0459 (6)	
C10	0.2030 (2)	0.8452 (6)	0.50522 (16)	0.0553 (8)	
C11	0.2834 (3)	0.9315 (6)	0.55060 (18)	0.0654 (10)	
H11	0.280349	1.071206	0.567794	0.078*	
C12	0.3664 (2)	0.8122 (6)	0.56994 (17)	0.0611 (9)	
H12	0.419441	0.873684	0.599499	0.073*	
C13	0.1282 (2)	0.5278 (6)	0.43591 (15)	0.0502 (7)	
H13	0.134933	0.386246	0.420945	0.060*	
C14	−0.0997 (7)	0.4207 (18)	0.4027 (6)	0.064 (2)	0.566 (17)
H14A	−0.128781	0.544663	0.417697	0.077*	0.566 (17)
H14B	−0.149973	0.335661	0.373353	0.077*	0.566 (17)
C15	−0.0319 (7)	0.510 (2)	0.3689 (4)	0.057 (2)	0.566 (17)
H15	−0.005627	0.382549	0.352299	0.069*	0.566 (17)
C16	−0.0758 (10)	0.669 (2)	0.3152 (4)	0.0572 (19)	0.566 (17)
C17	−0.0684 (10)	0.637 (2)	0.2553 (5)	0.066 (2)	0.566 (17)
H17	−0.039330	0.508395	0.247036	0.079*	0.566 (17)
C18	−0.1031 (9)	0.791 (2)	0.2075 (5)	0.074 (2)	0.566 (17)
H18	−0.096249	0.766965	0.168079	0.089*	0.566 (17)
C19	−0.1473 (8)	0.9778 (19)	0.2189 (5)	0.070 (2)	0.566 (17)
H19	−0.170095	1.082938	0.187252	0.084*	0.566 (17)
C20	−0.1585 (9)	1.011 (2)	0.2771 (6)	0.078 (2)	0.566 (17)
H20	−0.192024	1.134716	0.283963	0.093*	0.566 (17)
C21	−0.1202 (11)	0.863 (2)	0.3253 (5)	0.070 (2)	0.566 (17)
H21	−0.124083	0.893000	0.365174	0.084*	0.566 (17)
N3	0.04729 (18)	0.6253 (5)	0.41539 (12)	0.0517 (6)	0.566 (17)
O2	−0.0510 (6)	0.2833 (14)	0.4543 (5)	0.087 (3)	0.566 (17)
H2A	−0.089839	0.214465	0.466496	0.131*	0.566 (17)
C14A	−0.1148 (9)	0.483 (3)	0.3978 (8)	0.068 (3)	0.434 (17)
H14C	−0.114683	0.596685	0.428563	0.082*	0.434 (17)
H14D	−0.176288	0.484100	0.365402	0.082*	0.434 (17)
C15A	−0.0353 (9)	0.525 (3)	0.3690 (5)	0.058 (3)	0.434 (17)
H15A	−0.015932	0.382088	0.355769	0.070*	0.434 (17)
C16A	−0.0695 (12)	0.674 (3)	0.3122 (5)	0.059 (3)	0.434 (17)
C17A	−0.0522 (11)	0.607 (2)	0.2573 (6)	0.066 (3)	0.434 (17)
H17A	−0.020482	0.473357	0.256443	0.080*	0.434 (17)
C18A	−0.0823 (10)	0.738 (2)	0.2036 (4)	0.070 (3)	0.434 (17)
H18A	−0.070788	0.693030	0.166851	0.084*	0.434 (17)
C19A	−0.1298 (9)	0.937 (2)	0.2048 (5)	0.066 (3)	0.434 (17)
H19A	−0.149919	1.025228	0.168929	0.080*	0.434 (17)
C20A	−0.1470 (10)	1.005 (2)	0.2598 (6)	0.072 (3)	0.434 (17)
H20A	−0.178745	1.137755	0.260600	0.086*	0.434 (17)
C21A	−0.1169 (12)	0.873 (3)	0.3134 (5)	0.069 (3)	0.434 (17)
H21A	−0.128439	0.918086	0.350193	0.083*	0.434 (17)
N3A	0.04729 (18)	0.6253 (5)	0.41539 (12)	0.0517 (6)	0.434 (17)
O2A	−0.0972 (11)	0.2732 (18)	0.4266 (4)	0.093 (3)	0.434 (17)
H2B	−0.072975	0.288871	0.464641	0.140*	0.434 (17)
N1	0.5240 (2)	0.5375 (5)	0.60296 (15)	0.0645 (8)	
N2	0.4553 (2)	0.4536 (6)	0.56411 (14)	0.0628 (8)	
O1	0.12420 (19)	0.9622 (5)	0.48643 (15)	0.0800 (9)	
H1	0.084660	0.895236	0.458203	0.120*	
C22	0.8598 (3)	0.0019 (8)	0.6838 (2)	0.0820 (13)	
H22A	0.906649	0.083753	0.715575	0.123*	
H22B	0.883322	−0.026996	0.649024	0.123*	
H22C	0.846987	−0.136700	0.700952	0.123*	

### 2-{(E)-[(2-Hydroxy-1-phenylethyl)imino]methyl}-4-[(E)-(4-methylphenyl)diazenyl]phenol Atomic displacement parameters (Å^2^)

**Table:**

	*U*^11^	*U*^22^	*U*^33^	*U*^12^	*U*^13^	*U*^23^
C1	0.0540 (18)	0.059 (2)	0.069 (2)	0.0148 (16)	0.0266 (17)	0.0177 (17)
C2	0.064 (2)	0.061 (2)	0.072 (2)	0.0097 (18)	0.0208 (18)	0.0055 (18)
C3	0.056 (2)	0.074 (3)	0.071 (2)	0.0041 (18)	0.0172 (18)	0.009 (2)
C4	0.0563 (19)	0.064 (2)	0.070 (2)	0.0158 (18)	0.0288 (17)	0.0193 (19)
C5	0.063 (2)	0.063 (2)	0.072 (2)	0.0086 (18)	0.0259 (18)	0.0100 (19)
C6	0.0529 (19)	0.075 (3)	0.068 (2)	0.0034 (18)	0.0181 (17)	0.009 (2)
C7	0.0480 (16)	0.061 (2)	0.0542 (17)	0.0068 (14)	0.0177 (14)	0.0086 (15)
C8	0.0478 (16)	0.0506 (18)	0.0579 (18)	0.0077 (14)	0.0186 (14)	0.0008 (15)
C9	0.0434 (15)	0.0446 (16)	0.0511 (15)	0.0034 (13)	0.0168 (12)	0.0031 (13)
C10	0.0503 (18)	0.0476 (19)	0.0631 (19)	0.0072 (14)	0.0107 (15)	0.0028 (16)
C11	0.066 (2)	0.048 (2)	0.073 (2)	0.0039 (17)	0.0084 (18)	−0.0097 (18)
C12	0.0508 (18)	0.063 (2)	0.061 (2)	−0.0018 (16)	0.0057 (15)	−0.0031 (17)
C13	0.0519 (17)	0.0467 (17)	0.0565 (17)	0.0032 (14)	0.0232 (14)	0.0023 (14)
C14	0.052 (4)	0.062 (5)	0.080 (4)	0.002 (3)	0.021 (3)	−0.002 (4)
C15	0.048 (3)	0.064 (4)	0.061 (3)	−0.007 (3)	0.019 (3)	−0.008 (3)
C16	0.044 (3)	0.073 (4)	0.055 (3)	−0.009 (3)	0.015 (3)	−0.010 (3)
C17	0.056 (4)	0.078 (4)	0.064 (4)	−0.001 (3)	0.018 (3)	−0.010 (3)
C18	0.070 (4)	0.091 (5)	0.061 (3)	−0.009 (4)	0.019 (3)	−0.012 (4)
C19	0.066 (4)	0.084 (5)	0.058 (4)	−0.008 (4)	0.015 (4)	−0.006 (4)
C20	0.074 (4)	0.083 (4)	0.071 (5)	0.002 (4)	0.016 (4)	−0.002 (4)
C21	0.066 (4)	0.077 (4)	0.063 (4)	−0.001 (3)	0.014 (3)	−0.003 (4)
N3	0.0447 (12)	0.0554 (15)	0.0544 (13)	0.0008 (12)	0.0144 (11)	−0.0056 (13)
O2	0.056 (4)	0.102 (4)	0.093 (5)	−0.015 (3)	0.006 (4)	0.047 (4)
C14A	0.051 (4)	0.081 (6)	0.076 (5)	−0.007 (5)	0.024 (4)	0.010 (5)
C15A	0.049 (4)	0.062 (4)	0.062 (4)	−0.004 (4)	0.015 (4)	−0.011 (4)
C16A	0.045 (4)	0.073 (4)	0.059 (4)	−0.008 (4)	0.013 (4)	−0.011 (4)
C17A	0.058 (5)	0.083 (5)	0.058 (4)	−0.003 (4)	0.018 (4)	−0.013 (4)
C18A	0.069 (5)	0.082 (5)	0.059 (4)	−0.003 (4)	0.020 (4)	−0.019 (4)
C19A	0.066 (5)	0.080 (5)	0.054 (4)	0.000 (4)	0.020 (4)	−0.014 (4)
C20A	0.069 (4)	0.084 (4)	0.063 (5)	0.002 (4)	0.023 (4)	−0.008 (5)
C21A	0.065 (4)	0.080 (5)	0.060 (5)	0.004 (4)	0.016 (4)	−0.010 (4)
N3A	0.0447 (12)	0.0554 (15)	0.0544 (13)	0.0008 (12)	0.0144 (11)	−0.0056 (13)
O2A	0.093 (7)	0.115 (6)	0.054 (5)	−0.053 (5)	−0.005 (4)	0.018 (4)
N1	0.0606 (18)	0.0615 (19)	0.0714 (19)	0.0025 (15)	0.0206 (15)	0.0031 (16)
N2	0.0498 (15)	0.077 (2)	0.0577 (16)	0.0032 (14)	0.0118 (13)	0.0020 (16)
O1	0.0621 (16)	0.0603 (16)	0.098 (2)	0.0228 (14)	−0.0036 (14)	−0.0209 (15)
C22	0.061 (2)	0.086 (3)	0.101 (3)	0.027 (2)	0.029 (2)	0.021 (3)

### 2-{(E)-[(2-Hydroxy-1-phenylethyl)imino]methyl}-4-[(E)-(4-methylphenyl)diazenyl]phenol Geometric parameters (Å, º)

**Table:**

C1—C2	1.363 (5)	C16—C17	1.391 (8)
C1—C6	1.403 (5)	C17—C18	1.384 (9)
C1—N1	1.448 (4)	C17—H17	0.9300
C2—C3	1.373 (5)	C18—C19	1.361 (9)
C2—H2	0.9300	C18—H18	0.9300
C3—C4	1.380 (6)	C19—C20	1.377 (9)
C3—H3	0.9300	C19—H19	0.9300
C4—C5	1.389 (5)	C20—C21	1.376 (9)
C4—C22	1.502 (5)	C20—H20	0.9300
C5—C6	1.392 (5)	C21—H21	0.9300
C5—H5	0.9300	O2—H2A	0.8200
C6—H6	0.9300	C14A—O2A	1.400 (11)
C7—C8	1.369 (5)	C14A—C15A	1.526 (10)
C7—C12	1.405 (5)	C14A—H14C	0.9700
C7—N2	1.448 (4)	C14A—H14D	0.9700
C8—C9	1.394 (4)	C15A—N3A	1.463 (9)
C8—H8	0.9300	C15A—C16A	1.507 (8)
C9—C10	1.422 (5)	C15A—H15A	0.9800
C9—C13	1.432 (4)	C16A—C17A	1.3900
C10—O1	1.312 (4)	C16A—C21A	1.3900
C10—C11	1.400 (5)	C17A—C18A	1.3900
C11—C12	1.370 (5)	C17A—H17A	0.9300
C11—H11	0.9300	C18A—C19A	1.3900
C12—H12	0.9300	C18A—H18A	0.9300
C13—N3A	1.281 (4)	C19A—C20A	1.3900
C13—N3	1.281 (4)	C19A—H19A	0.9300
C13—H13	0.9300	C20A—C21A	1.3900
C14—O2	1.419 (10)	C20A—H20A	0.9300
C14—C15	1.526 (8)	C21A—H21A	0.9300
C14—H14A	0.9700	O2A—H2B	0.8200
C14—H14B	0.9700	N1—N2	1.221 (4)
C15—N3	1.475 (7)	O1—H1	0.8200
C15—C16	1.517 (8)	C22—H22A	0.9600
C15—H15	0.9800	C22—H22B	0.9600
C16—C21	1.387 (8)	C22—H22C	0.9600
			
C2—C1—C6	120.7 (3)	C16—C17—H17	119.0
C2—C1—N1	115.4 (4)	C19—C18—C17	119.4 (8)
C6—C1—N1	123.9 (4)	C19—C18—H18	120.3
C1—C2—C3	119.7 (4)	C17—C18—H18	120.3
C1—C2—H2	120.1	C18—C19—C20	120.2 (8)
C3—C2—H2	120.1	C18—C19—H19	119.9
C2—C3—C4	121.5 (4)	C20—C19—H19	119.9
C2—C3—H3	119.2	C21—C20—C19	120.2 (8)
C4—C3—H3	119.2	C21—C20—H20	119.9
C3—C4—C5	118.9 (3)	C19—C20—H20	119.9
C3—C4—C22	120.3 (4)	C20—C21—C16	121.1 (8)
C5—C4—C22	120.8 (4)	C20—C21—H21	119.5
C4—C5—C6	120.4 (4)	C16—C21—H21	119.5
C4—C5—H5	119.8	C13—N3—C15	119.0 (6)
C6—C5—H5	119.8	C14—O2—H2A	109.5
C5—C6—C1	118.7 (4)	O2A—C14A—C15A	106.6 (10)
C5—C6—H6	120.6	O2A—C14A—H14C	110.4
C1—C6—H6	120.6	C15A—C14A—H14C	110.4
C8—C7—C12	118.5 (3)	O2A—C14A—H14D	110.4
C8—C7—N2	115.1 (3)	C15A—C14A—H14D	110.4
C12—C7—N2	126.4 (3)	H14C—C14A—H14D	108.6
C7—C8—C9	122.1 (3)	N3A—C15A—C16A	109.9 (9)
C7—C8—H8	119.0	N3A—C15A—C14A	110.6 (10)
C9—C8—H8	119.0	C16A—C15A—C14A	110.7 (10)
C8—C9—C10	119.0 (3)	N3A—C15A—H15A	108.5
C8—C9—C13	119.7 (3)	C16A—C15A—H15A	108.5
C10—C9—C13	121.3 (3)	C14A—C15A—H15A	108.5
O1—C10—C11	120.2 (3)	C17A—C16A—C21A	120.0
O1—C10—C9	121.3 (3)	C17A—C16A—C15A	117.9 (8)
C11—C10—C9	118.5 (3)	C21A—C16A—C15A	122.1 (8)
C12—C11—C10	120.7 (3)	C18A—C17A—C16A	120.0
C12—C11—H11	119.6	C18A—C17A—H17A	120.0
C10—C11—H11	119.6	C16A—C17A—H17A	120.0
C11—C12—C7	121.2 (3)	C17A—C18A—C19A	120.0
C11—C12—H12	119.4	C17A—C18A—H18A	120.0
C7—C12—H12	119.4	C19A—C18A—H18A	120.0
N3A—C13—C9	122.5 (3)	C20A—C19A—C18A	120.0
N3—C13—C9	122.5 (3)	C20A—C19A—H19A	120.0
N3—C13—H13	118.7	C18A—C19A—H19A	120.0
C9—C13—H13	118.7	C21A—C20A—C19A	120.0
O2—C14—C15	111.1 (7)	C21A—C20A—H20A	120.0
O2—C14—H14A	109.4	C19A—C20A—H20A	120.0
C15—C14—H14A	109.4	C20A—C21A—C16A	120.0
O2—C14—H14B	109.4	C20A—C21A—H21A	120.0
C15—C14—H14B	109.4	C16A—C21A—H21A	120.0
H14A—C14—H14B	108.0	C13—N3A—C15A	123.1 (8)
N3—C15—C16	108.1 (7)	C14A—O2A—H2B	109.5
N3—C15—C14	108.4 (7)	N2—N1—C1	112.5 (3)
C16—C15—C14	115.3 (8)	N1—N2—C7	113.1 (3)
N3—C15—H15	108.3	C10—O1—H1	109.5
C16—C15—H15	108.3	C4—C22—H22A	109.5
C14—C15—H15	108.3	C4—C22—H22B	109.5
C21—C16—C17	117.1 (7)	H22A—C22—H22B	109.5
C21—C16—C15	120.6 (7)	C4—C22—H22C	109.5
C17—C16—C15	122.2 (8)	H22A—C22—H22C	109.5
C18—C17—C16	121.9 (8)	H22B—C22—H22C	109.5
C18—C17—H17	119.0		

### 2-{(E)-[(2-Hydroxy-1-phenylethyl)imino]methyl}-4-[(E)-(4-methylphenyl)diazenyl]phenol Hydrogen-bond geometry (Å, º)

**Table:**

*D*—H···*A*	*D*—H	H···*A*	*D*···*A*	*D*—H···*A*
O2—H2*A*···O1^i^	0.82	2.00	2.745 (10)	151
O1—H1···N3	0.82	1.88	2.605 (4)	147
O2*A*—H2*B*···O2^ii^	0.82	2.14	2.88 (2)	149

Symmetry codes: (i) −*x*, *y*−1, −*z*+1; (ii) −*x*, *y*, −*z*+1.
